# The relationship between greenspace and the mental wellbeing of adults: A systematic review

**DOI:** 10.1371/journal.pone.0203000

**Published:** 2018-09-12

**Authors:** Victoria Houlden, Scott Weich, João Porto de Albuquerque, Stephen Jarvis, Karen Rees

**Affiliations:** 1 Warwick Institute for Science of Cities, University of Warwick, Coventry, West Midlands, United Kingdom; 2 ScHARR, University of Sheffield, Sheffield, South Yorkshire, United Kingdom; 3 Centre for Interdisciplinary Methodologies, University of Warwick, Coventry, West Midlands, United Kingdom; 4 Department of Computer Science, University of Warwick, Coventry, West Midlands, United Kingdom; 5 Warwick Medical School, University of Warwick, Coventry, West Midlands, United Kingdom; CUNY, UNITED STATES

## Abstract

**Introduction:**

The view that interacting with nature enhances mental wellbeing is commonplace, despite a dearth of evidence or even agreed definitions of ‘nature’. The aim of this review was to systematically appraise the evidence for associations between greenspace and mental wellbeing, stratified by the different ways in which greenspace has been conceptualised in quantitative research.

**Methods:**

We undertook a comprehensive database search and thorough screening of articles which included a measure of greenspace and validated mental wellbeing tool, to capture aspects of hedonic and/or eudaimonic wellbeing. Quality and risk of bias in research were assessed to create grades of evidence. We undertook detailed narrative synthesis of the 50 studies which met the review inclusion criteria, as methodological heterogeneity precluded meta-analysis.

**Results:**

Results of a quality assessment and narrative synthesis suggest associations between different greenspace characteristics and mental wellbeing. We identified six ways in which greenspace was conceptualised and measured: (i) amount of local-area greenspace; (ii) greenspace type; (iii) visits to greenspace; (iv) views of greenspace; (v) greenspace accessibility; and (vi) self-reported connection to nature. There was adequate evidence for associations between the amount of local-area greenspace and life satisfaction (hedonic wellbeing), but not personal flourishing (eudaimonic wellbeing). Evidence for associations between mental wellbeing and visits to greenspace, accessibility, and types of greenspace was limited. There was inadequate evidence for associations with views of greenspace and connectedness to nature. Several studies reported variation in associations between greenspace and wellbeing by life course stage, gender, levels of physically activity or attitudes to nature.

**Conclusions:**

Greenspace has positive associations with mental wellbeing (particularly hedonic wellbeing), but the evidence is not currently sufficient or specific enough to guide planning decisions. Further studies are needed, based on dynamic measures of greenspace, reflecting access and uses of greenspace, and measures of both eudaimonic and hedonic mental wellbeing.

## Introduction

### Background

Urbanisation is increasing at an unprecedented rate, and with over half the world’s population now residing in cities [1], many people may not have access to the green landscapes in which the human species evolved [2, 3]. Greenspace may provide human benefits, such as facilitating exercise, social activities and connecting with nature [4], and it is suggested that urban greenspaces are critical to healthy living, both physically [5, 6] and mentally [7, 8]. There may also be salutogenic effects on mental health and wellbeing, such as increased attention, feelings of happiness and reduced stress [9, 10].

The *United Nations Sustainable Development Goals* emphasise the importance of greenspace provision “to foster prosperity and quality of life for all” [11]. The World Health Organisation stated that urban greenspaces (including parks, woodlands, and sports facilities) are a “necessary component for delivering healthy, sustainable, liveable conditions” [12], while highlighting the dearth of evidence to support planning advice [12]. In the UK, local authorities are responsible for providing access to the natural environment [13], and guidelines recommend that all residents should live within 300m of at least 2 hectares of greenspace [14, 15], despite limited evidence for the wellbeing benefits of these recommendations.

### Measuring greenspace

One of the reasons for this dearth of evidence is the lack of consensus regarding the definition of the terms ‘nature’ and ‘natural’ [10, 16], and features that may appear ‘natural’ are often artificially constructed [8]. Hartig et al. provide the most detailed definition of nature, as the “physical features and processes of nonhuman origin…, the ‘living nature’ of flora and fauna” [8].

Furthermore, ‘nature’ and ‘greenspace’ are often used interchangeably [17–21]; ‘greenspace’ is more inclusive, referring to areas of grass, trees or other vegetation [22], and can be used to describe both surrounding greenness in the countryside, and spaces managed or reserved in urban environments [14]. Greenspace was therefore chosen as the focus of this review. We chose not to include studies of water (blue space), as this is generally considered separately to greenspace [5, 23–25].

### Mental wellbeing and greenspace

Mental wellbeing comprises happiness and life satisfaction (hedonic wellbeing) and fulfilment, functioning and purpose in life (eudaimonic wellbeing) [26, 27]. It is therefore a multi-dimensional measure of positive mental health, reflecting more than an absence of mental distress, in which those with the best mental wellbeing are able to realise their potential, cope well with everyday stressors, and flourish mentally. It is increasingly recognised as an indicator of national prosperity [28], due to its associations with productivity, longevity and societal functioning [28–30]. While theories suggest that mental wellbeing may be improved by exposure to greenspace, there is limited evidence for clear benefits; many studies use unvalidated measures or proxies such as mental distress or quality of life [7]. Additionally, measures of nature and greenspace vary widely [8, 12, 22].

Previous reviews have examined the relationship between greenspace (/nature) and general health [7, 8, 12], or mental health [31], although the latter has generally been defined in terms of mental distress, rather than mental wellbeing. While Douglas et al. describe their recent scoping review as focussing on “green space benefits for health and well-being”, they include no studies measuring mental wellbeing per se, but provide further evidence for reduced mental distress in greener neighbourhoods [7]. Similarly, Gascon et al.’s review of “Mental Health Benefits” of long-term greenspace exposure includes some studies of aspects of mental wellbeing, but focusses mainly on measures of mental distress, rather than positive mental health [31]. We therefore believe this is the first review to examine greenspace associations specifically with mental wellbeing, in adults.

The aim of this review was therefore to synthesise quantitative evidence for associations between greenspace and mental wellbeing. We were able to identify varying evidence for associations between different characterisations of greenspace and mental wellbeing, while highlighting key areas for future research, and subsequent implications for policy and practice.

## Materials and methods

### Search strategy and selection criteria

The review was registered with PROSPERO (available at https://www.crd.york.ac.uk/prospero/, ID: CRD42016041377). We followed guidance from York’s Centre for Research and Dissemination and the Cochrane Handbook for Systematic Reviews [32, 33]. A search strategy was developed with an information specialist, undertaken by one reviewer (VH), supported by a second, independent reviewer (SW). The following databases were searched: Applied Social Sciences Index and Abstracts (ASSIA), American Psychological Association (PsychInfo), National Center for Biotechnology Information (PubMED), Elsevier’s Scopus, and Web of Science (WOS). Common keywords relating to greenspace and mental wellbeing were derived from the literature, refined following a trial search in each database; this created a final set of terms for greenspace (greenspace(s), green space(s), open space(s), green, greener, nature, natural, landscape) and mental wellbeing (wellbeing, well-being, wellbeing, happiness, happy, happier, life satisfaction, satisfaction with life). We restricted searches to studies in English, relating to humans, published after 01/01/1980. Searches were run from 07/07/2016 to 31/01/2018. The full electronic searches are shown in Table 1.

**Table 1:** Database search strategy.

*Database*	*Search*
ASSIA	ti(green?space OR "open space" OR green* OR natur* OR landscape) AND ti(wellbeing OR well?being OR "mental health" OR happy OR happi* OR life NEAR/5 satisfaction)
PubMed	(((((((greenspace[Title] OR "green space"[Title] OR "open space"[Title] OR green*[Title] OR nature[Title] OR natural[Title] OR landscape[Title])) AND (well-being[Title] OR wellbeing[Title] OR "well being"[Title] OR "mental health"[Title] OR happy[Title] OR happier[Title] OR happiness[Title] OR "life satisfaction"[Title])) AND ("1980/01/01"[PDat]: "2018/01/31"[PDat]) AND Humans[Mesh] AND English[lang])))
PsychInfo	ti(green?space OR "open space" OR green* OR natur* OR landscape) AND ti(wellbeing OR well?being OR "mental health" OR happy OR happi* OR life NEAR/5 satisfaction) AND la.exact("English")
Scopus	((TITLE (greenspace OR (open space) OR (green space) OR green OR greener OR nature OR natural OR landscape) AND TITLE (well?being OR wellbeing OR (mental health) OR happy OR happier OR happiness OR (life W/5 satisfaction)))) AND PUBYEAR > 1979) AND ORIG-LOAD-DATE AFT 1529266261 AND ORIG-LOAD-DATE BEF 1529871076 AND PUBYEAR AFT 2016 AND (LIMIT-TO (LANGUAGE, "English"))
WOS	TITLE: (("green space*" OR greenspace* OR "open space*" OR greener OR green OR nature OR natural OR landscape)) <i>AND</i> TITLE: ((well?being OR wellbeing OR "mental health" OR happy OR happiness OR happier OR life NEAR/5 satisfaction)) Refined by: *LANGUAGES:* (ENGLISH)

Using the in-built database functions, an auto-search was timed to re-run each query on a weekly basis to detect any further publications within the review duration. All articles recovered from initial searches were recorded in Endnote, and duplicates removed. Titles and Abstracts were screened for potential relevance by two reviewers independently, and full texts of shortlisted studies retrieved for formal inclusion/exclusion. It was agreed that any disputed studies would be cautiously retained for full text evaluation.

### Study eligibility criteria

Criteria for inclusion were: (a) Population: adults aged over 16 (or all ages, but not wholly or mainly children); (b) Exposure: any measure of greenspace, defined as areas of grass, trees or other vegetation. Studies measuring personal connectedness to nature were included. As we were interested in all greenspace characteristics, we included both urban and rural studies; (c) Control: Comparators must include a control group which differed in the type/degree of exposure to greenspace, or direct comparison before and after an intervention; (d) Outcome: mental wellbeing, ascertained using a validated measure of hedonic and/or eudaimonic mental wellbeing, or one or more aspects of these (e.g. life satisfaction, happiness, quality of life. The General Health Questionnaire (GHQ) is designed to measure psychological distress, but includes several positive items, and is prevalent in the literature; studies using this outcome were therefore included. Instruments designed to capture only symptoms of mental distress were not included; (e) No study designs were explicitly excluded.

### Evaluation of evidence

After identifying eligible papers, one reviewer (VH) evaluated study contents by extracting: authors, publication date, country, study design, age of participants, sample size, greenspace measures, methods, outcomes, confounders, and a results summary, including effect sizes (regression coefficients/risk ratio and confidence interval/standard error).

For quality appraisal, risk of bias was assessed using Cochrane-recommended criteria [32]: the Newcastle-Ottawa Scale (NOS), adapted for longitudinal and cross-sectional studies, alongside the Cochrane Risk of Bias (RoB) tool for controlled studies [34, 35]. The criteria cover potential risk of bias arising from: representativeness of the sample, participant awareness of the intervention, control factors, and selection of reported results.

We used established Quality Assessment thresholds to categorise each article [36]. For those assessed using the Cochrane RoB tool, a Good quality study met all criteria (low RoB), while those of Fair quality had moderate RoB not meeting one criterion; Poor quality studies had high RoB, not meeting multiple criteria. More complex scoring criteria were used for papers analysed using the NOS, across three domains: Selection (representativeness of sample, treatment of non-respondents), Comparability (between exposure groups) and Outcome (assessment, soundness). Good studies scored at least 3 for Selection, 1 for Comparability and 2 for Outcome; Fair studies scored at least 2, 1 and 2, respectively. Poor papers scored 1 or less for each category. A final quality rating was given according to the lowest rating for any category.

### Stratification by characterisation of greenspace

We identified six types of study, according to the characterisation of greenspace: (a) amount of local-area greenspace, most commonly the proportion of local areas covered by greenspace; (b) greenspace type; (c) views of greenspace; (d) visits to greenspace; (e) accessibility, in terms of proximity to greenspaces and self-reported ‘access’; and (f) subjective connection to nature.

We conducted a narrative review of evidence, as methodological heterogeneity precluded meta-analysis. Evidence for associations between each type of greenspace characteristic and mental wellbeing was classified according to the consistency, strength and methodological quality of the findings, and study design. Evidence of association was categorised using established guidelines used by other studies in the field [37]: *Adequate* (most studies, at least one Good quality, reported an association between greenspace and mental wellbeing); *Limited* (more than one study, at least one Good, reported an association, but with inconsistent findings); *Inadequate* (associations reported in one or more studies, but none Good quality); and *No association* (several Good quality studies reported an absence of a statistically significant association between greenspace and mental wellbeing).

## Results

**Fig 1:**
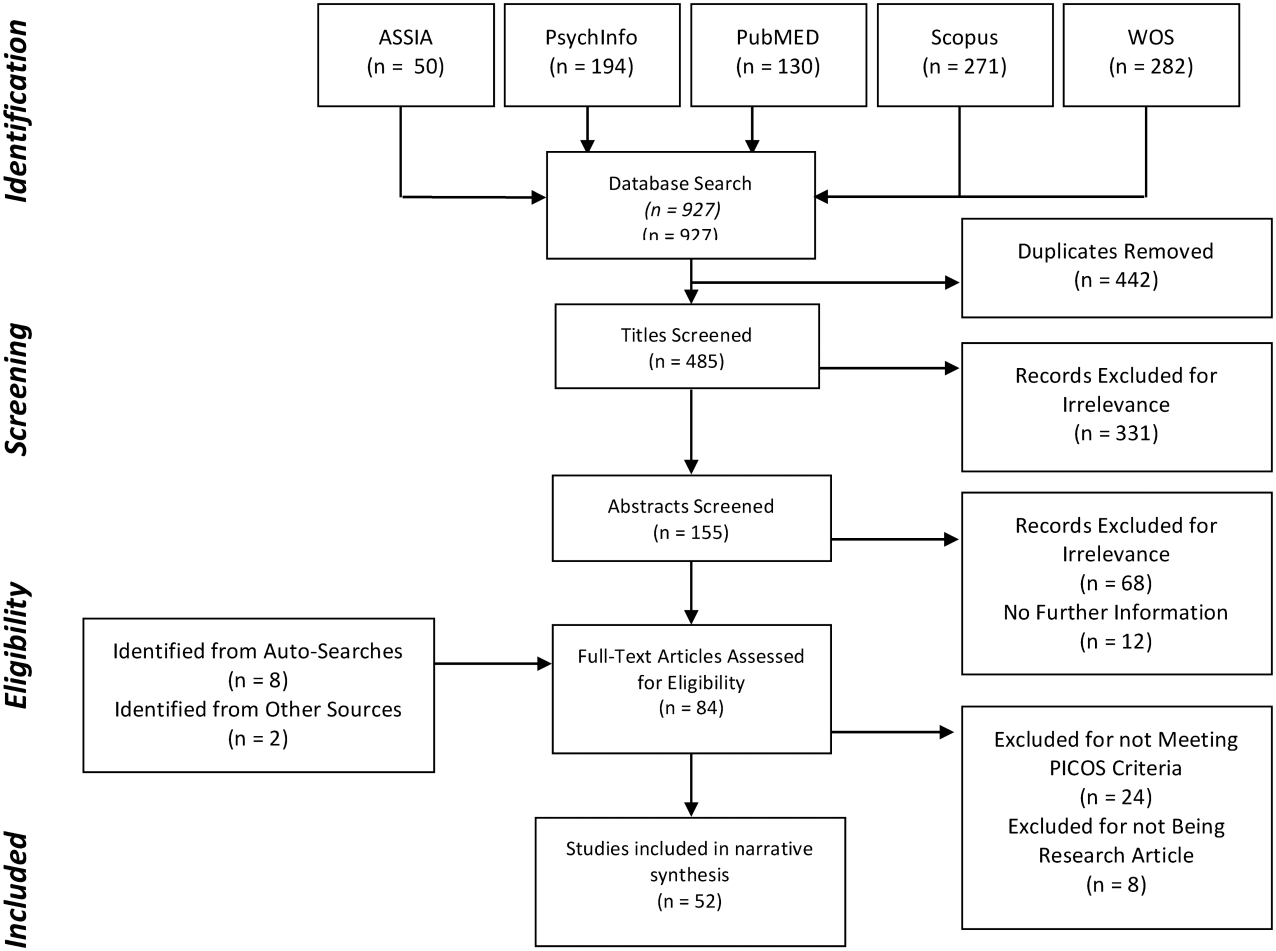
Study selection process.

Titles and abstracts of 485 records were screened, and 75 chosen for full-text evaluation; 42 were found to be eligible. During this process, 10 additional papers were found via Auto-Searching the databases and recommendations. Therefore, 52 papers were finally included in this review (Fig 1).

Among these, 4 were controlled case studies and a further 6 were longitudinal cohort studies; there was one ecological analysis, 4 uncontrolled case studies, the remaining 37 were cross-sectional surveys. Two studies were international, 31 were restricted to Europe, 15 just in the UK; 5 were based in the USA with another 6 in Canada, 10 in Australia. Analyses were confined to urban areas in 22 cases, 9 included only rural greenspace. Sample size ranged from 25 to 30,900 participants, but was not specified in 3 cases. Age ranges were fairly consistent, covering young adults to past retirement age, although 1 focused on ‘youths’ (aged 16–25), 3 studies recruited university students and two included mainly people aged over 55; however, 11 studies did not specify participants’ age. After quality assessment, the majority of studies (*n* = 27) were determined to be Good, 13 were Fair, and 12 Poor. For Poor studies, Table 2 provides further justification. For full details of the risk of bias for each study, heat maps are presented in S1 and S2 Tables. Table 3 provides further detail on the typologies of greenspace measures implemented for each study.

**Table 2:** Main characteristics and results of included studies.

*Authors*, *Year*, *Country*	*Study Design*	*Age of Participants*	*Sample Size*	*Greenspace Measure*		*Mental Wellbeing Tool*	*Mental Wellbeing*	*Confounders*	*Methods*	Statistically Significant Associations**	Effect Size** (C: *Correlation Coefficient*, *SE*: *Standard Error*, *CI*: *Confidence Interval)*	*Interaction Effects*	*Quality*
***a) Amount of Local- Area Greenspace***													
Alcock et al., 2015, England [38]	Longitudinal Cohort Study	under 25- over 75	2,020 214 movers	% area of each LSOA*, 10 land-cover types *Rural areas only*		GHQ-12	Psychological Distress	Individual: Demographic, Marital, SES, Living Conditions, Health Commuting. Local: IMD	Multilevel Linear Regression	Cross-sectional differences: no association. Longitudinal differences for movers: significant, positive associations with increase access individually to Arable, Improved Grassland, Semi-natural Grassland, Mountain, Heath and Bog, and Coastal land cover.	C, SE: Within-individual: Arable: 0.083, 0.037 Improved Grassland: 1.351, 0.040 Semi-natural Grassland: 0.152, 0.062 Mountains/Heath: 1.667, 0.074	N/A	Good
Alcock et al., 2014, England [23]	Longitudinal Cohort Study	16–55+	1,064 residents of BHPS who relocated during survey	% greenspace in each LSOA, including private gardens, *Urban areas only*		GHQ-12	Psychological Distress	Individual: Demographic, Marital, SES, Living Conditions, Health, Pre-move GHQ, Commuting. Local: IMD	Linear Regression	Movers to greener areas: significantly lower GHQ scores post-move. Movers to less green areas: GHQ decreased in year preceding the move but no significant difference post-move.	C, SE: Movers to greener areas T+1: 0.369, 0.152 T+2: 0.378, 0.158 T+3: 0.431, 0.162	N/A	Good
Ambrey and Fleming, 2014, Australia [39]	Cross-Sectional Survey	15–60+	NOT GIVEN	% public greenspace in each CD* Urban areas only		Life Satisfaction	Life Satisfaction	Individual: Demographic, Language, Marital, SES, Living Conditions, Health, Commuting, Hours Worked	Linear Regression	*More greenspace*: *higher life satisfaction*	C, SE: 0.003, 0.002	N/A	Good
Ambrey, 2016, Australia [40]	Cross-Sectional Survey	NOT GIVEN	3,288	Greenspace per capita, in each CD *Urban areas only*		SF-36 Mental Component Survey	Mental Health	Individual: Physical Activity	Linear Regression	More greenspace: better mental health, only for those engaged in physical activity	C, SE: Greenspace Physical Activity Interaction: 4.392, 1.702	Positive interaction between greenspace and physical activity	Good
Ambrey, 2016, Australia [41]	*Cross-Sectional Survey*	NOT GIVEN	6,082	Greenspace per capita, in each CD *Urban areas only*		Life Satisfaction, SF-36	Life Satisfaction, Quality of Life	Individual: Physical Activity	Logistic Regression	More greenspace: better life satisfaction and quality of life	Odds, CI: Life Satisfaction: 0.942, 0.920–0.990. Quality of Life: 0.974, 0.912–1.039	N/A	Good
Ambrey, 2016, Australia [42]	Cross-Sectional Survey	NOT GIVEN	6,077	Amount of greenspace in each CD *Urban areas only*		SF-36	Quality of Life	Individual: Demographic, Ethnicity, Marital, SES, Free Time, Social Interaction, Household Members Engaged in Physical Activity, Personality. Local: Proximity to Lake, River, Coastline, SES	*Linear Regression*	More greenspace: better quality of life, only for those engaged in physical activity	C, SE: 0.553, 0.229	Positive interaction between greenspace and physical activity	Good
Astell-Burt et al., 2014, UK [17]	Longitudinal Cohort Study	15–75+	65,407 person-years	% greenspace in each ward, excluding water and private gardens *Urban areas only*		GHQ-12	Psychological Distress	Individual: Demographic, Marital, SES, Living Conditions, Smoking	Linear Regression	More greenspace: lower GHQ scores among men. Variation in associations across life course and gender.	C, SE: ‘High’ Greenspace: 0.300, 0.370	Interactions for life course and gender	Good
Bos et al., 2016, The Netherlands [43]	Cross-Sectional Survey	18–87	4,924	% greenspace within 1km and 3km buffers		Manchester Short Assessment of Quality of Life	Quality of Life	Individual: Demographic, Country of Origin, Marital, SES	Linear Regression	More greenspace within 3km: better quality of life, significant interactions for age and gender. For middle aged men, inverse association Greenspace within 1km: no association	C, SE: 1km: 5.200, 5.500. 3km: 6.300, 4.500	Interactions for life course and gender	Poor Limited Statistical reporting
De Vries et al., 2003, The Netherlands [5]	Cross-Sectional Survey	All ages (including children)	10,179	% greenspace in local area, % bluespace in local area, presence of a garden		GHQ-12	Psychological Distress	Individual: Demographic, SES, Living Conditions, Health Insurances, Life Events in Last Year	Multilevel Linear Regression	More greenspace: lower GHQ scores Access to agricultural space: lower GH Only for lower educated groups Results only significant for whole sample, not for individual urban categories Having a garden: significant only in very urban municipalities	C, SE: %green within 3km: -0.100, 0.003	Interaction with level of urbanity	Good
De Vries et al., 2013, The Netherlands [44]	Cross- Sectional Survey	NOT GIVEN	1,641	Quantity and quality of streetscape greenery, *Urban areas only*		SF-36	Quality of Life	Individual: Demographic, SES, Living Conditions, Health, Life Events in Last Year,	Multilevel Linear Regression	Higher amounts of greenspace: higher QOL, but not statistically significant after quality is added to the model. High quality of greenspace: higher quality of life.	C, SE: Quantity: 0.007, 0.036 (not statistically significant) Quality: 0.0153, 0.069	Both Quantity and Quality show positive interactions with stress, social cohesion, and green activity	Good
Dzhambov et al., 2018, Bulgaria [45]	Cross- Sectional Survey	15–25	399	Amount of green land within 500m of home, perceived neighbourhood greenness and quality *Urban areas only*		GHQ-12	Psychological Distress	Individual: Demographic, SES, Living Conditions, Noise. Local: Population Density	Linear Mixed Models and Linear Mediation Models	Perceived greenness and quality: lower GHQ scores. No statistically significant associations for objective greenspace measures.	C, CI: Perceived greenness: -0.59, -0.85- -0.32 Greenspace quality: -0.08, -0.12 - -0.04	Higher perceived restorative quality was associated with more physical activity and social cohesion, which was associated with lower GHQ scores. For objective measures, this held for all but the greenspace quality measure.	Fair
Houlden et al., 2017, England [46]	Cross-Sectional Survey	16–65+	30,900	% greenspace in each LSOA, excluding gardens		SWEMWBS	Mental Wellbeing	Individual: Demographic, Marital, SES, Living Conditions, Health, Commuting. Local: IMD	Linear Regression	Greater amounts of greenspace: higher SWEMWBS scores. Reduced to null after adjustment	No statistically significant associations to report	N/A	Good
Maas et al., 2009, The Netherlands [47]	Cross-sectional Survey	12–65+	10,089	%greenspace within 1 and 3km buffers		GHQ-12	Psychological Distress	Individual: Demographic, Ethnicity, SES, Living Conditions, Health Insurance, Life Events in Last Year. Local: Level of Urbanity	Multilevel Linear Regression	More surrounding greenspace: lower GHQ score. Stronger association for 1km than 3km	C, SE: 1km: -0.005, 0.002 3km: -0.004, 0.002	N/A	Good
Taylor et al., 2018, Australia and New Zealand [48]	Cross-Sectional Survey	18–75+	1,819	Amount of greenspace in postcode *Urban areas only*		WHO-5	Hedonic Wellbeing	NO	Linear Regression	Higher amounts of greenspace: higher WHO-5 scores. Only for 2 sample cities, remaining 2 insignificant	C: Melbourne: 1.410 Sydney: 2.470	N/A	Poor No controls
Triguero-Mas et al., 2015, Spain [49]	Cross-Sectional Survey	NOT GIVEN	8,793	Amount of greenspace within 300m buffer Sensitivity analysis with other buffers		GHQ-12	Psychological Distress	Individual: Demographic, Birth Place, Marital, SES, Health Insurance. Local: SES	Logistic Regression	Higher amounts of greenspace: lower odds of higher GHQ score Consistent results for all buffers	Odds, CI: Males: 0.820, 0.700–0.980 Females: 0.770, 0.670–0.880	Stronger association for males than females	Fair
Triguero-Mas et al., 2017, Europe [50]	Cross-Sectional Survey	18–75	403	Amount of greenspace within 300m buffer, *Urban areas only*		SF-36 Mental Component Survey	Mental Health	Individual: Demographic	Linear Regression	No association for surrounding greenspace.	No Statistical Results to report	Stronger association for males than females	Fair
Vemuri and Costanza, 2006, International [51]	Ecological Analysis	NOT GIVEN	172 Countries	Ecosystem services product (ESP), per square kilometre for each country, normalised. From amount of each land-cover and multiplied by ecosystem services per country.		Life Satisfaction	Life Satisfaction	NO	Linear Regression	Better natural capital: higher life satisfaction	Odds, SE: 2.453, 0.739	N/A	Poor No controls, high-level analysis
Ward Thompson et al., 2014, Scotland [52]	Cross-Sectional Survey	NOT GIVEN	305	Amount of greenspace “around each home”, perceptions of local greenspace, *Urban areas only*		SWEMWBS	Mental Wellbeing	Individual: Demographic, Income, Deprivation	Linear Regression	Perceptions of having sufficient local greenspace: better mental wellbeing Satisfaction with quality: higher mental wellbeing	No Statistical Results to Report	N/A	Fair
White et al., 2013, England [24]	Cross-Sectional Survey	Under 25-over75	12,818 (GHQ) 10,168 (Life Satisfaction)	% greenspace in each LSOA, including private gardens, *Urban areas only*		Life Satisfaction, GHQ	Life Satisfaction, Psychological Distress	Individual: Demographic, Marital, SES, Living Conditions, Health, Commuting. Local: IMD	Linear Regression	Higher percentage of greenspace: decreased GHQ, increased Life Satisfaction	C, SE: GHQ: -0.004, 0.001 Life Satisfaction: 0.002, 0.001	N/A	Good
White et al., 2013, England [25]	Cross-Sectional Survey	Under 25-over75	15,361	% greenspace in each LSOA, including private gardens		Life Satisfaction, GHQ	Life Satisfaction, Psychological Distress	Individual: Demographic, Marital, SES, Living Conditions, Health, Commuting. Local: IMD	Linear Regression	Higher percentage of greenspace: decreased GHQ	C, SE: GHQ (reversed): Greenspace: 0.003, 0.001	N/A	Good
Wood et al., 2017, Australia [53]	Cross-Sectional Survey	NOT GIVEN	492	Amount and number of public greenspaces within 1.6km buffer, type of greenspace: sports, recreational, natural *Urban areas only*		SWEMWBS	Mental Wellbeing	Individual: Demographic, SES	Linear Regression	Number of parks: higher mental wellbeing. Strongest association for largest parks, decreasing with size. Greater area of parks: higher mental wellbeing scores Strongest association for sports spaces	C, SE: Number of parks: 0.110, 0.050 Hectare increase of park area: 0.070, 0.020 Number of sports spaces: 0.430, 0.210 Number of recreational spaces: 0.110, 0.050 Number of natural spaces: 0.110, 0.050	N/A	Fair
***b) Greenspace Types***													
Alcock et al., 2015, England [38]	Longitudinal Cohort Study	under 25- over 75	2,020 *214 movers*	10 land-cover types *Rural areas only*		GHQ-12	Psychological Distress	Individual: Demographic, Marital, SES, Living Conditions, Health Commuting. Local: IMD	Multilevel Linear Regression	Cross-sectional differences: no association. Longitudinal differences for movers: significant, positive associations with increase access individually to Arable, Improved Grassland, Semi-natural Grassland, Mountain, Heath and Bog, and Coastal land cover.	C, SE: Within-individual: Arable: 0.083, 0.037 Improved Grassland: 1.351, 0.040 Semi-natural Grassland: 0.152, 0.062 Mountains/Heath: 1.667, 0.074	N/A	Good
Annerstedt et al., 2012, Sweden [54]	Longitudinal Cohort Study	18–80	7,549 residents who did not relocate during survey	Presence of 5 green qualities within 300m buffer: Serene, Wild, Lush, Spacious, Culture *Rural areas only*		GHQ-12	Psychological Distress	Individual: Demographic, Country of Origin, Marital, Financial Strain, Physical Activity	Logistic Regression	Presence of Serene: lower GHQ score, only for those engaged in physical activity Presence of Spacious: lower GHQ, only for women engaged in physical activity	*Odds*, *CI*: *Women with Access to Serene*: *0*.*200*, *0*.*060–0*.*900*	Positive interaction between being physical activity and serene greenspace Positive interaction between being physical activity and serene greenspace, for women	*Good*
Bjork et al., 2008, Sweden [18]	Cross-Sectional Survey	19–76	24,819	Number of 5 green qualities within 100 and 300m buffers: Serene, Wild, Lush, Spacious, Culture *Rural areas only*		SF-36 Vitality Component Survey	Vitality	Individual: Demographic, SES, Financial Strain, Smoking	Logistic Regression	More green qualities within 300m: better vitality, only for women More green qualities within 100m: no association Individual qualities: no association	Odds and CI, women with access to number of qualities: 4–5: 1.070, 0.880–1.290 3: 1.220, 1.060–1.410 2: 1.060,0.940–1.190	Interactions with gender	Good
Luck et al., 2011, Australia [55]	Cross-sectional Survey	All ages	1,043	Residential neighbourhood greenspace aspects:, vegetation cover, vegetation density, *Urban areas only*		Subjective Wellbeing	Subjective Wellbeing	Individual: Demographic, SES, Living Conditions, General Activity	Multilevel Linear Regression	Higher levels of species richness, species abundance, vegetation cover, vegetation density: better subjective wellbeing, strongest for vegetation	C, SE: Vegetation Cover: 0.560, 0.260 Vegetation Density: 0.800, 0.390	N/A	Good
MacKerron and Mourato, 2013, UK [56]	Cross-Sectional Survey	All ages	21,947	Land cover types		Happiness	Happiness	NO	Linear Regression	All outdoor land cover types: better happiness than continuous urban areas. Marine and coastal areas have happiest scores.	C, SE: Mountains/moors: 2.710, 0.870 Woodland: 2.120, 0.340 Semi-natural grassland: 2.040, 0.350 Suburban/rural: 0.880, 0.160	N/A	Fair
Sugiyama et al., 2008, Australia [57]	Cross-Sectional Survey	20–65	1,895	Neighbourhood Environment Walkability Scale, *Urban areas only*		SF-36 Mental Component Survey	Mental Health	Individual: Demographic, Marital, SES, Walking, Social Interaction	Logistic Regression	Higher reported greenness: better mental health	Odds, CI: High Perceived Greenness: 1.270, 0.990–1.620	N/A	Good
Van den Bosch et al., 2015, Sweden [58]	Longitudinal Cohort Study	18–80	1,419 residents who relocated during survey	Amount and presence of greenspace within 300m buffer: Serene, Wild, Lush, Spacious, Culture, *Rural areas only*		GHQ-12	Psychological Distress	Individual: Deprivation, Marital, Education	Logistic Regression	Gained access to Serene greenspace: improved mental health among women. No other associations	Odds, CI: Access to Serene: 2.800, 1.110–7.040	Associations only for females, not males	Good
Vemuri et al., 2011, USA [59]	Cross-sectional Survey	18–65+	1,361	Neighbourhood satisfaction, quality of neighbourhood natural environment, amount of tree cover per census block, *Urban areas only*		Life Satisfaction	Life Satisfaction	Individual: Demographic, Ethnicity, Marital, Living Conditions, Social Capital	Logistic Regression	Stronger perceived environmental quality: improved life satisfaction Perceived shows stronger association than objective measures	C, SE: 0.276, 0.514	N/A	Good
Weimann et al., 2015, Sweden [60]	Longitudinal Cohort Study	18–80	9,444	Number of 5 green qualities within local 1km^2^ area: Serene, Wild, Lush, Spacious, Culture		GHQ-12	Psychological Distress	Individual: Demographic, Marital, SES, Living Conditions BMI, Smoking	Multilevel Logistic Regression	Within-individual difference of higher neighbourhood greenness: lower psychological distress	Odds, CI: Within-Individual: 1.030, 1.000–1.160 Between-Individuals:1.070, 1.000–1.140	N/A	Good
Wood et al., 2017, Australia [53]	Cross-Sectional Survey	NOT GIVEN	492	Amount and number of public greenspaces within 1.6km buffer, type of greenspace: sports, recreational, natural *Urban areas only*		SWEMWBS	Mental Wellbeing	Individual: Demographic, SES	Linear Regression	Number of parks: higher mental wellbeing. Strongest association for largest parks, decreasing with size. Greater area of parks: higher mental wellbeing scores Strongest association for sports spaces	C, SE: Number of parks: 0.110, 0.050 Hectare increase of park area: 0.070, 0.020 Number of sports spaces: 0.430, 0.210 Number of recreational spaces: 0.110, 0.050 Number of natural spaces: 0.110, 0.050	N/A	Fair
***c) Views of Greenspace***													
Gilchrist et al., 2015, Scotland [61]	Cross-Sectional Survey	16–55+	366	Workplace view naturalness, view satisfaction, extent of features in view *Urban areas only*		SWEMWBS	Mental Wellbeing	Individual: Demographic, Job Type, Greenspace Use in Leisure Time. Local: Location	Linear Regression	No association for view naturalness Satisfaction with view, views of trees/bushes/flowering plants: higher SWEMWBS score Types strongest predictors	C, SE: View of Trees: 0.616, 0.198 View bushes/flowers: 0.610, 0.312 View Satisfaction: 0.802, 0.215	N/A	Good
Pretty et al., 2005, UK [20]	Controlled Case Study	18–60	100	Running while exposed to photographs: urban/rural pleasant and unpleasant		Rosenberg Self-Esteem Questionnaire, Profile of Mood States	Self-Esteem, Mood	*NO*	N/A	Viewing pleasant scenes: increase in self-esteem	No Statistical Results to Report	N/A	Fair
Vemuri et al., 2011, USA [59]	Cross-sectional Survey	18–65+	1,361	Number of trees visible from residence *Urban areas only*		Life Satisfaction	Life Satisfaction	Individual: Demographic, Ethnicity, Marital, Living Conditions, Social Capital	Logistic Regression	Perceived shows stronger association than objective measures	No Statistical Results to Report	N/A	Good
***d) Visits to Greenspace***													
Duvall and Kaplan, 2014, USA [62]	Uncontrolled Case Study	20–50+	73	Wilderness Expedition, *Rural areas only*		AFI, PANAS	Attention, Affect	Individual: Demographic, SES, Physical and Mental Health History, Veteran History	Linear Mixed Models	Post expedition: more positive affect and better attentional functioning Follow-up: better positive affect	Score Change: AFI: 0.340 Affect: 0.270	N/A	Poor Small sample, allocation based on intervention
Dzhambov et al., 2018, Bulgaria [45]	Cross- Sectional Survey	15–25	399	Amount of green land within 500m of home, Euclidean distance to nearest greenspace, perceived neighbourhood greenness and quality, travel time to and time spent in neighbourhood greenspace *Urban areas only*		GHQ-12	Psychological Distress	Individual: Demographic, SES, Living Conditions, Noise. Local: Population Density	Linear Mixed Models and Linear Mediation Models	Perceived greenness and quality, and travel time to greenspace: lower GHQ scores. No statistically significant associations for objective greenspace measures.	C, CI: Perceived greenness: -0.59, -0.85- -0.32 <5min to greenspace: -2.54, -3.96 - -1.12 Greenspace quality: -0.08, -0.12 - -0.04	Higher perceived restorative quality was associated with more physical activity and social cohesion, which was associated with lower GHQ scores. For objective measures, this held for all but the greenspace quality measure.	Fair
Gilchrist et al., 2015, Scotland [61]	Cross-Sectional Survey	16–55+	366	Workplace greenspace visit frequency, weekly use duration *Urban areas only*		SWEMWBS	Mental Wellbeing	Individual: Demographic, Job Type, Greenspace Use in Leisure Time. Local: Location	Linear Regression	No association for use frequency Time spent in workplace greenspace, satisfaction with view, views of trees/bushes/flowering plants: higher SWEMWBS score Types strongest predictors	C, SE: Use Duration: 0.431, 0.191	N/A	Good
Herzog and Stevey, 2008, USA [63]	Cross-Sectional Survey	University Students	823	Self-reported typical contact with nature		Ryff’s Scales of Psychological Well-Being, Attention, PANAS	Mental Wellbeing, Attention, Affect	Individual: Sense of humour	Linear Regression	Greater contact with nature: better personal development, effective functioning.	C: Personal Development: 0.090 Effective Functioning: 0.230	N/A	Fair
Jakubec et al., 2016, Canada [64]	Uncontrolled Case Study	Adults	37	Visits to greenspace, *Rural areas only*		Quality of Life Inventory	Quality of Life	NO	*Score Change*	Post-Intervention: improved quality of life, not statistically significant	Score Change: Satisfaction with love: +1.000 Satisfaction with life: -1.000	N/A	Poor No controls, participants aware of intervention
Kamitsis and Francis, 2013, Australia [65]	Cross-Sectional Survey	18–69	190	Nature Exposure, CNS		WHOQOL-BREF	Quality of Life	Individual: Spirituality	Linear Regression	Higher nature exposure or connection to nature: better quality of life	C: Exposure: 0.280 CNS: 0.330	N/A	Poor Minimal controls
Marselle et al., 2013, UK [66]	Controlled Case Study	Adults, mostly over 55	708	Group walks in different environments: natural and semi-natural, green corridors, farmland, parks and gardens, urban, coastal, amenity greenspace, allotments, outdoor sports facilities, other		WEMWBS, PANAS	Mental Wellbeing, Affect	Individual: Demographic, Marital, Education, Deprivation	Multilevel Linear Regression	Walks in farmland: better mental wellbeing No associations with other greenspace types	C, SE: Walks in farmland: 2.790, 0.003	N/A	Fair
Marselle et al., 2015, UK [67]	Cross-Sectional Survey	Adults, mostly over 55	127	Walking: environment type, perceived naturalness, perceived biodiversity, perceived restorativeness, duration of walk, perceived walk intensity		Happiness, PANAS	Happiness, Affect	*NO*	Multilevel Linear Regression	Perceived restorativeness, perceived walk intensity: positively associated with affect and happiness.	C, SE: Affect: 0.126, 0.014 Happiness: 0.029, 0.003	N/A	Poor No controls, participants aware of intervention
Mitchell, 2013, Scotland [19]	Cross-sectional Survey	16+	1,890	Frequency of use of different environment types for physical activity		WEMWBS, GHQ	Mental Wellbeing, Psychological Distress	Individual: Demographic, Income, Physical Activity. Local: Level of Urbanity	Linear Regression	Regular use of open space/park or woods/forest: lower GHQ score Regular use of natural environments: no clear association with mental wellbeing Regular use of non-natural environments: better mental wellbeing	Odds, CI: GHQ: Park >1 a week: 0.570, 0.369–0.881 Woods >1 a week: 0.557, 0.323–0.962 WEMWBS: Park <1 a week: 2.442, 0.769–4.115	N/A	Good
Molsher and Townsend, 2016, Australia [68]	Uncontrolled Case Study	14–71	32	Engagement with 10 week Environmental Volunteering Project, *Rural areas only*		General Wellbeing Scale, PANAS	Wellbeing, Affect	NO	Score Change	Post-intervention and Follow-up: improved wellbeing and mood state scores	Score Change: Wellbeing: +11.600	N/A	Poor No controls, participants aware of intervention
Nisbet and Zekenski, 2011, Canada [69]	Controlled Case Study	16–48	150	Walking indoors or outdoors in nature, Nature Relatedness *Urban areas only*		Happiness, PANAS	Happiness, Affect	NO	T-Tests	Walking outdoors: more positive affect, relaxation and fascination	T-Test: Outdoor Walk: Affect: 4.860 Relaxation: 4.570 Fascination: 4.800	N/A	Fair
Panno et al., 2017, Italy [70]	Cross-Sectional Survey	NOT GIVEN	115	Self-reported greenspace visit frequency		WHO-5	Hedonic Wellbeing	Individual: Demographics, SES	Hierarchical Regression	Higher reported frequency of greenspace visits: greater wellbeing scores. Not statistically significant.	No Statistically Significant Results to Report	N/A	Fair
Richardson et al., 2016, UK [71]	Uncontrolled Case Study	18–71	613	Nature in Self, Engagement with “30 Days Wild” Programme		Happiness	Happiness	NO	T-Tests	Post-intervention, increased nature connection, increased general happiness	T-Tests: 6.650	N/A	Fair
Triguero-Mas et al., 2017, Europe [50]	Cross-Sectional Survey	18–75	403	Frequency of contact with greenspace in terciles *Urban areas only*		SF-36 Mental Component Survey	Mental Health	Individual: Demographic	Linear Regression	Lower frequency of greenspace visits: poorer mental health. Stronger associations for males	C, CI for “low” contact Males: -9.140, -14.420 - -3.860 Females: -5.000, -9.790- -0.021	Stronger association for males than females	Fair
Van den Berg et al., 2016, Spain, The Netherlands, Lithuania, UK [21]	Cross-Sectional Survey	18–75	3,748	Reported hours of greenspace visits in last month, *Urban areas only*		SF-36 Mental Component Survey	Mental Health	Individual: Demographic, SES, Living Conditions, Childhood Nature Experience	Multilevel Linear Regression	Higher visits to greenspace: better mental health	C, CI: 0.030, 0.020–0.040	N/A	Good
Ward Thompson et al., 2014, Scotland [52]	Cross-Sectional Survey	NOT GIVEN	305	Patterns of greenspace use *Urban areas only*		SWEMWBS	Mental Wellbeing	Individual: Demographic, Income, Deprivation	Linear Regression	No association between greenspace use and mental wellbeing	No Statistical Results to Report	N/A	Fair
White et al., 2017, England [72]	Cross-Sectional Survey	NOT GIVEN	7,272	Did the individual visit greenspace yesterday. Amount of time spent outdoors *Urban areas only*		ONS4	Mental Wellbeing	Individual: Demographic, Marital, SES, Living Conditions, Health, Commuting. Local: IMD	Logistic Regression	Visiting a greenspace yesterday: higher happiness Spending time outdoors: more frequently associated with higher worth, decreasing with frequency	C, CI: Visited greenspace yesterday, happiness: 1.660, 1.320–2.080 Spending time outdoors everyday day, compared to never, worth: 1.960, 1.490–2.580	N/A	Good
***e) Greenspace Accessibility***													
Bjork et al., 2008, Sweden [18]	Cross-Sectional Survey	19–76	24,819	Number of 5 green qualities within 100 and 300m buffers: Serene, Wild, Lush, Spacious, Culture *Rural areas only*		SF-36 Vitality Component Survey	Vitality	Individual: Demographic, SES, Financial Strain, Smoking	Logistic Regression	More green qualities within 300m: better vitality, only for women More green qualities within 100m: no association Individual qualities: no association	Odds and CI, women with access to number of qualities within 300m: 4–5: 1.070, 0.880–1.290 3: 1.220, 1.060–1.410 2: 1.060,0.940–1.190	Interactions with gender	Good
Bos et al., 2016, The Netherlands [43]	Cross-Sectional Survey	18–87	4,924	% greenspace within 1km and 3km buffers		Manchester Short Assessment of Quality of Life	Quality of Life	Individual: Demographic, Country of Origin, Marital, SES	Linear Regression	More greenspace within 3km: better quality of life, significant interactions for age and gender. For middle aged men, inverse association Greenspace within 1km: no association	C, SE: 1km: 5.200, 5.500. 3km: 6.300, 4.500	Interactions for life course and gender	Poor Limited Statistical reporting
Dadvand et al., 2016, Spain [73]	Cross-Sectional Survey	18–65+	3461	% greenspace within 100m, 250m and 500m buffers, subjective presence of greenspace within 10 minute walk, objective presence of greenspace within 200m of minimum 5000m^2^ *Urban areas only*		GHQ-12	Psychological Distress	*Individual*: *Demographic*, *SES*, *Social Support*, *Physical Activity* *Local*: *Deprivation*	*Logistic Regression*	*More greenspace nearer to home*: *lower GHQ score*. *Effect sizes decreasing with distance*. *Greater subjective and objective proximity to greenspace*: *lower GHQ scores*	*Odds*, *CI*: *100m*: *1*.*320*, *1*.*160–1*.*510* *250m*: *1*.*250*, *1*.*100–1*.*400* *500m*: *1*.*170*, *1*.*040–1*.*320* *Subjective proximity*: *1*.*300*, *1*.*040–1*.*630* *Objective proximity*: *1*.*200*, *0*.*970–1*.*480*	*N/A*	Good
Dzhambov et al., 2018, Bulgaria [45]	Cross- Sectional Survey	15–25	399	Amount of green land within 500m of home, Euclidean distance to nearest greenspace, perceived neighbourhood greenness and quality, travel time to greenspace *Urban areas only*		GHQ-12	Psychological Distress	Individual: Demographic, SES, Living Conditions, Noise. Local: Population Density	Linear Mixed Models and Linear Mediation Models	Travel time to greenspace: lower GHQ scores. No statistically significant associations for objective greenspace measures.	C, CI: <5min to greenspace: -2.54, -3.96 - -1.12	Lower travel time to greenspace was associated with more physical activity and social cohesion, which was associated with lower GHQ scores..	Fair
Krekel et al., 2015, Germany [74]	Cross-sectional Survey	17–99	NOT GIVEN	Euclidean distance from home to green and abandoned areas *Urban areas only*		SF-36 Mental Component Survey	Mental Health	Individual: Demographic, Country of Origin, Marital, SES, Living Conditions, Disabilities	Linear Regression	Access to urban greenspaces: better mental health Access to abandoned areas: poorer mental health	C: Greenspace: 0.007	N/A	Good
Maas et al., 2009, The Netherlands [47]	Cross-sectional Survey	12–65+	10,089	%greenspace within 1 and 3km buffers		GHQ-12	Psychological Distress	Individual: Demographic, Ethnicity, SES, Living Conditions, Health Insurance, Life Events in Last Year. Local: Level of Urbanity	Multilevel Linear Regression	More surrounding greenspace: lower GHQ score. Stronger association for 1km than 3km	C, SE: 1km: -0.005, 0.002 3km: -0.004, 0.002	N/A	Good
Sugiyama et al., 2008, Australia [57]	Cross-Sectional Survey	20–65	1,895	Neighbourhood Environment Walkability Scale, *Urban areas only*		SF-36 Mental Component Survey	Mental Health	Individual: Demographic, Marital, SES, Walking, Social Interaction	Logistic Regression	Higher reported greenness: better mental health	Odds, CI: High Perceived Greenness: 1.270, 0.990–1.620	N/A	Good
Triguero-Mas et al., 2015, Spain [49]	Cross-Sectional Survey	NOT GIVEN	8,793	Amount of greenspace within 100m, 300m, 500m and 1km buffers, presence of green and blue spaces within buffer Sensitivity analysis with other buffers		GHQ-12	Psychological Distress	Individual: Demographic, Birth Place, Marital, SES, Health Insurance. Local: SES	Logistic Regression	Higher amounts of greenspace: lower odds of higher GHQ score Consistent results for all buffers	Odds, CI: Males: 0.820, 0.700–0.980 Females: 0.770, 0.670–0.880	Stronger association for males than females	Fair
***f) Subjective Connection to Nature***													
Cervinka et al., 2012, Austria [75]	Cross-Sectional Survey	15–87	547		CN-SI*	SF-36 Component Surveys, SWLS, WHOQOL-BREF	Quality of Life, Life Satisfaction	Individual: Demographic	Linear Regression	Higher CN-SI Score: better meaningfulness, mental health, vitality and emotional-role function	C: Meaningfulness: 0.210 Mental Health: 0.180 Vitality: 0.230 Emotions: 0.190	N/A	Poor *Limited sampling description*
Howell et al., 2011, Canada [76]	Cross-Sectional Survey	University Students	452		CNS*	Keyes’ Index of Well-Being and Mindful Attention Awareness Scale	Mental Wellbeing, Attention	NO	Linear Regression	Greater connection to nature: greater psychological wellbeing and social wellbeing. Not associated with emotional wellbeing or mindfulness	C: Psychological Wellbeing: 0.150 Social Wellbeing: 0.200	N/A	Poor No controls, minimal reporting
Howell et al., 2013, Canada [77]	Cross-Sectional Survey	University Students	311		CNS, Nature Relatedness Scale*	Emotional Wellbeing, Steen Happiness Index, Meaning in Life Questionnaire, Meaningful Life Measure, General Life Purpose Scale	Mental Wellbeing, Happiness, Meaning in Life	NO	Linear Regression	Greater connection to nature: better reported wellbeing, meaning in life	C: Meaning: 0.310 Purpose: 0.250 Happiness: 0.220 Emotional Wellbeing: 0.200 Psychological Wellbeing: 0.250 Social Wellbeing: 0.260	N/A	Poor *No controls*, *minimal reporting*
Kamitsis and Francis, 2013, Australia [65]	Cross-Sectional Survey	18–69	190		Nature Exposure, CNS	WHOQOL-BREF	Quality of Life	Individual: Spirituality	Linear Regression	Higher nature exposure or connection to nature: better quality of life	C: Exposure: 0.280 CNS: 0.330	N/A	Poor Minimal controls
Nisbet et al., 2011, Canada [78]	Cross-Sectional Survey	Adults, student subgroup	184, 145,in two studies		Nature Relatedness Scale, New Ecological Consciousness Scale	Ryff’s Psychological Well-Being Inventory, SWLS, PANAS	Mental Wellbeing, Life Satisfaction, Affect	NO	Linear Regression	Higher nature relatedness: better wellbeing, positive affect, purpose in life. No association for life satisfaction.	C: Study 1: Affect: 0.330 Purpose: 0.230 Study 2: Affect: 0.220 Purpose: 0.240	N/A	Fair
Zelenski et al., 2014, Canada [79]	Cross-Sectional Survey	NOT GIVEN	950		Nature Relatedness Scale, Inclusion of Nature in Self	Ryff’s PWBI, SWLS, Subjective Happiness Scale (SHS), PANAS	Mental Wellbeing, Life Satisfaction, Happiness, Affect	NO	Linear Regression	Stronger connection to nature: improved wellbeing, happiness, life satisfaction and affect	C: Wellbeing: 0.250 Happiness: 0.360 Life Satisfaction: 0.310 Affect: 0.380	N/A	Poor No controls
Zhang et al., 2014, USA [80]	Cross-Sectional Survey	18–88	1,108		CNS, Engagement with Natural Beauty Scale	SWLS	Life Satisfaction	Individual: Demographic, Personality	Multilevel Linear Regression	Higher connectedness with nature: improved life satisfaction, only for those reporting being attuned to nature’s beauty	C, CI: Connectedness: 0.1000, -0.990–0.109 Engagement: 0.155, 0.121–0.344 ConnectednessXENGAGEMENT: 0.080, 0.170–0.151	Positive interaction between connectedness to nature and being attuned to nature’s beauty	Good

*LSOA, Lower-Layer Super Output Area, a census-based spatial unit. CD, Census District, a census-based spatial unit.

*CNS, Connectedness to Nature Scale, measure of individuals’ trait levels of feeling emotionally connected to the natural world. CN-SI, single-item version of CNS. Nature Relatedness Scale, affective, cognitive, and experiential aspects of individual’s connection to nature

**All associations described in this table are statistically significant, unless otherwise specified

**Table 3:** Greenspace measures employed in included studies.

Study	Greenspace Type	Measure Type	Metrics Used	Spatial Scale
***a) Amount of Local-Area Greenspace***				
Alcock et al., 2015 [38]	Natural Land Cover	Land Cover Map	Proportion of area that is greenspace	LSOA
Alcock et al., 2014 [23]	Greenspace and Private Gardens	Generalised Land Use Database (GLUD)	Proportion of area that is greenspace	LSOA
Ambrey and Fleming, 2014 [39]	Public Greenspace (including public parks, community gardens cemeteries, sports fields, national parks and wilderness areas)	GIS	Proportion of area that is greenspace	Census District
Ambrey, 2016 [40]	Public Greenspace (including public parks, community gardens cemeteries, sports fields, national parks and wilderness areas)	GIS	Amount of greenspace per Capita	Census District
Ambrey, 2016 [41]	Public Greenspace (including public parks, community gardens cemeteries, sports fields, national parks and wilderness areas)	GIS	Amount of greenspace per Capita	Census District
Ambrey, 2016 [42]	Public Greenspace (including public parks, community gardens cemeteries, sports fields, national parks and wilderness areas)	GIS	Amount of greenspace per Capita	Census District
Astell-Burt et al., 2014 [17]	Green and Natural Environment (excluding water and private gardens)	Land Use Database	Proportion of area that is greenspace	Ward
Bos et al., 2016 [43]	Greenspace (urban green including vegetable gardens, sports areas >0.5ha, parks >1ha; and rural green including agricultural and natural green)	Dutch Land Use Database and GIS	Proportion of area that is greenspace	1km and 3km buffers of postcode centroid
De Vries et al., 2003 [5]	Greenspace (urban green, agricultural green, forests and nature areas)	National Land Use Classification Database and GIS	Proportion of area that is greenspace	3km around centre of neighbourhood unit
De Vries et al., 2013 [44]	All types of visible vegetation, and quality based on variation, maintenance, orderly arrangement, absence of litter and general impression of greenspace	On-street Audit	Level of greenness (1- the street does not make a very green impression, to 5- the street makes a very green impression)	Average street greenness of neighbourhood unit
Dzhambov et al., 2018 [45]	Green land cover	NDVI	Proportion of area that is greenspace	500m Euclidean buffer of home
	Greenspace (parks, gardens, street trees)	Self-reported	Perceived neighbourhood greenness and quality, travel time to and time spent in neighbourhood greenspace, green views from home	Self-reported neighbourhood
Houlden et al., 2017 [46]	Greenspace	Generalised Land Use Database (GLUD)	Proportion of area that is greenspace	LSOA
Maas et al., 2009 [47]	Greenspace (urban green, agricultural green, forests and nature areas)	National Land Use Classification Database and GIS	Proportion of area that is greenspace	1km and 3km buffer around individual’s home
Taylor et al., 2018 [48]	Green land cover	NDVI	NDVI value	Postcode
Triguero-Mas et al., 2015 [49]	Green land cover	NDVI	Amount of greenspace	300m Euclidean buffer of postcodes
Triguero-Mas et al., 2017 [50]	Green land cover	NDVI	Amount of greenspace	300m Euclidean buffer of postcodes
Vemuri and Costanza, 2006 [51]	Land Cover Types	Land Cover Map	Ecosystem Services Product (amount of each land cover, multiplied by ecosystem services per country)	Country
Ward Thompson et al., 2014 [52]	Greenspace (parks, woodlands, scrub and other publicly accessible natural environments)	GIS	Amount of Greenspace	Neighbourhood unit
White et al., 2013 [24]	Greenspace and Private Gardens	Generalised Land Use Database (GLUD)	Proportion of area that is greenspace	LSOA
White et al., 2013 [25]	Greenspace and Private Gardens	Generalised Land Use Database (GLUD)	Proportion of area that is greenspace	LSOA
Wood et al., 2017 [53]	Greenspace (parks and other areas of green public open spaces)	Land Cover Map	Amount and number of parks	1.6km road network buffer
***b) Greenspace Types***				
Alcock et al., 2015 [38]	Land Cover Types (broadleaf woodland, coniferous woodland, arable, improved grassland, semi-natural grassland, mountain, heath and bog, saltwater, freshwater, coastal, built-up areas including gardens)	Land Cover Map	Proportion of area of each type	LSOA
Annerstedt et al., 2012 [54]	5 qualities: Serene (place of peace, silence and care), Wild (place of fascination with wild nature), Lush (place rich in species), Spacious (place offering a restful feeling of entering another world), Culture (the essence of human culture)	CORINE Land Cover and GIS	Presence of each type	3km Euclidean buffer from home
Bjork et al., 2008 [18]	5 qualities: Serene, Wild, Lush, Spacious, Culture	CORINE Land Cover and GIS	Presence of each type	100 and 300m Euclidean buffers from home
Luck et al., 2011 [55]	Vegetation Cover (woody and non-woody vegetation)	Advanced Land Observation Satellite	Proportion of vegetation	Census District
	Vegetation Density (understory, mid-story and over-story cover)	Field Survey	Proportion of vegetation	Census District
MacKerron and Mourato, 2013 [56]	Land Cover Classes (marine and coastal, freshwater and wetlands, mountains and moors and heathland, semi-natural grasslands, farmland, coniferous woodland, broadleaf woodland, bare ground, suburban/rural development, continuous urban)	Land Cover Map	Type	Current GPS location
Sugiyama et al., 2008 [57]	Neighbourhood Greenness	Self-Reported	Level of greenness	Neighbourhood unit
Van den Bosch et al., 2015 [58]	5 qualities: Serene, Wild, Lush, Spacious, Culture	CORINE Land Cover and GIS	Amount and presence of each type	300m Euclidean buffer from home
Vemuri et al., 2011 [59]	Natural environment quality and satisfaction	Self-Reported	Perceptions of neighbourhood	Neighbourhood
Weimann et al., 2015 [60]	5 qualities: Serene, Wild, Lush, Spacious, Culture	CORINE Land Cover and GIS	Presence of each type	5–10 minute walk from homes
Wood et al., 2017 [53]	Sports, recreational, and natural green spaces	Land Cover Map	Amount and presence of each type	1.6km network buffer of homes
**Views of Greenspace**				
Gilchrist et al., 2015 [61]	Workplace greenspace	Self–Reported	Perceptions of view of greenspace naturalness and extent	Workplace
Pretty et al., 2005 [20]	Rural pleasant and unpleasant scenes Urban pleasant and unpleasant scenes	Lab environment setting	Photographs	Photographs of views
Vemuri et al., 2011 [59]	Number of trees visible from home	Self-Reported	Perceptions of neighbourhood	Individual
**c) *Visits to Greenspace***				
Duvall and Kaplan, 2014 [62]	Wilderness	Objective	Exposure through expedition	Individual
Dzhambov et al., 2018 [45]	Parks and gardens	Self-Reported	Time spent in greenspace	Self-reported Neighbourhood
Gilchrist et al., 2015 [61]	Workplace greenspace	Self–Reported	Frequency and duration of greenspace exposure	Workplace
Herzog and Stevey, 2008 [63]	Nature	Self-Reported	Typical contact	Individual
Jakubec et al., 2016 [64]	Wilderness	Objective	Exposure through expedition	Individual
Kamitsis and Francis, 2013 [65]	Nature	Self-Reported	Level of exposure	Individual
Marselle et al., 2013 [66]	Natural and semi-natural, green corridors, farmland, parks/gardens, urban, coastal, amenity green space, allotments, outdoor sports facilities, other	Land Use Database	Walking while exposed to different environments	Individual
Marselle et al., 2015 [67]	Natural and semi-natural, green corridors, farmland, parks/gardens, urban, coastal, amenity green space, allotments, outdoor sports facilities, other	Land Use Database,	Duration of walk and environment type	Individual
	Natural and semi-natural, green corridors, farmland, parks/gardens, urban, coastal, amenity green space, allotments, outdoor sports facilities, other	Self-Reported	Perceived naturalness, biodiversity, restorativeness, walk intensity	Individual
Mitchell, 2013 [19]	Woodland/forest, open space/park, country paths, beach/river, sports field/courts, swimming pool, gym/sports centre, pavements, home/garden, other, none	Self-Reported	Frequency of use of different greenspace types for physical activity	Individual
Molsher and Townsend, 2016 [68]	Rural nature	Objective	Engagement with 10-week Environmental Volunteering Project	Individual
Nisbet and Zekenski, 2011 [69]	Outdoors (in nature)	Objective	Walking indoors vs outdoors	Individual
Panno et al., 2017 [70]	Greenspace	Self-Reported	Greenspace visit frequency	Individual
Richardson et al., 2016 [71]	Nature	Self-Reported	Engagement with 100 days wild programme	Individual
Triguero-Mas et al., 2017 [50]	Natural outdoor environment	Urban Atlas, CORINE Land Cover and GIS	Duration of exposure to nature	Individual
Van den Berg et al., 2016 [21]	Greenspace (Public and private open spaces that contain “green” and/or “blue” natural elements such as street trees, forests, city parks and natural parks/reserves)	Self-Reported	Duration of visits to greenspace	Individual
Ward Thompson et al., 2014 [52]	Greenspace (parks, woodlands, scrub and other publicly accessible natural environments)	Self-Reported	Frequency of greenspace visits	Individual
White et al., 2017 [72]	Greenspace	Self-Reported	Having visited a greenspace yesterday	Individual
***d) Greenspace Accessibility***				
Bjork et al., 2008 [18]	5 qualities: Serene, Wild, Lush, Spacious, Culture	CORINE Land Cover and GIS	Presence of each type	100 and 300m Euclidean buffer of home
Bos et al., 2016 [43]	Greenspace (urban green including vegetable gardens, sports areas >0.5ha, parks >1ha; and rural green including agricultural and natural green)	Dutch Land Use Database and GIS	Proportion of area that is greenspace	1km and 3km Euclidean buffers of postcode centroid
Dadvand et al., 2016 [73]	Green land cover	NDVI	Proportion of area that is greenspace Presence of 5000m^2^ greenspace within 200m	100m, 250m and 500m Euclidean buffer of home
	Greenspace	Self-Reported	Proximity to greenspace	10 minute walk from home
Dzhambov et al., 2018 [45]	Greenspace (park, allotment, or recreational grounds)	OpenStreetMap and GIS	Proximity to greenspace	Euclidean distance from home
Krekel et al., 2015 [74]	Urban green areas (greens, forests, and waters), and abandoned urban areas	European Urban Atlas	Proximity to greenspace	Euclidean distance from home
Maas et al., 2009 [47]	Greenspace (urban green, agricultural green, forests and nature areas)	National Land Use Classification Database and GIS	Proportion of area that is greenspace	1km and 3km Euclidean buffer of home
Sugiyama et al., 2008 [57]	Neighbourhood Greenness	Self-Reported	Access to park or nature reserve	Neighbourhood
Triguero-Mas et al., 2015 [49]	Green land cover	NDVI	Amount of greenspace	100m, 300m, 500m, 1km Euclidean buffer of home
***e) Subjective Connection to Nature***				
Cervinka et al., 2012 [75]	Nature	Self-Reported	Connectedness to nature	Individual
Howell et al., 2011 [76]	Nature	Self-Reported	Connectedness to nature	Individual
Howell et al., 2013 [77]	Nature	Self-Reported	Connectedness to nature Nature relatedness	Individual
Kamitsis and Francis, 2013 [65]	Nature	Self-Reported	Connectedness to nature	Individual
Nisbet et al., 2011 [78]	Nature	Self-Reported	Nature relatedness Ecological consciousness	Individual
Zelenski et al., 2014 [79]	Nature	Self-Reported	Nature relatedness Inclusion of nature in self	Individual
Zhang et al., 2014 [80]	Nature	Self-Reported	Connectedness to nature Engagement with natural beauty	Individual

### Mental wellbeing measures

Only 14 studies were found to measure both hedonic and eudaimonic mental wellbeing, of which the most commonly used measure was the Warwick-Edinburgh Mental Well-Being Scale (WEMWBS) [19, 46, 52, 53, 61, 66]. WEMWBS includes 14 positively worded questions, regarding individual feelings over the past 2 weeks, including “feeling relaxed”, “interested in new things”, and “close to others” [81]; there is also a reduced 7-item version, known as SWEMWBS (Shortened-WEMWBS) [82]. The recent Personal Wellbeing ONS4, applied in to one study [72], measures individuals’ life satisfaction, happiness and anxiety (hedonic wellbeing) and sense of worth (eudaimonic wellbeing) [83].

The remaining 32 studies assessed outcomes considered to be aspects of mental wellbeing, such as quality of life, life satisfaction, and affect, but did not report both hedonic and eudaimonic wellbeing. The WHO-5 Well-Being Index, used in 2 studies [48, 70], asks how frequently individuals have felt “cheerful and in good spirits” and “calm and relaxed”, over the previous 2 weeks, but focusses on hedonic rather than eudaimonic wellbeing [84].

Quality of life was measured in 6 studies, two using the WHOQOL-BREF [65, 75], a 26-item questionnaire covering physical and psychological health, social relationships and personal environment [85]. The SF-36 instrument measures quality of life with 36 physical, emotional and psychological health questions [86], and was used in 4 studies [41, 42, 44, 75]. A brief 12-item version (SF-12) has three subscales: mental health, vitality [18], and emotional-role functioning. The mental component summary (MCS), derived from a subset of emotional problems, wellbeing and social functioning questions, was used in 6 papers [21, 40, 50, 57, 74, 75], asking how often the individual recently felt “full of energy”, “nervous” and “happy” [86].

Single-item Life Satisfaction was used in 6 studies [24, 25, 40, 41, 51, 87]. The Satisfaction With Life Scale (SWLS) was applied to 4 studies [75, 78–80], and includes a more thorough 5 life-evaluation questions, which ask how ideal and satisfying the individual’s life is, and if they have “gotten the important things… in life” [88].

Happiness was measured with one question in 4 studies [56, 67, 69, 71]. The Attentional Functioning Index (AFI), which assesses daily functioning, was used in one study [62, 89].

Eight studies reported affect scores [62, 63, 66–69, 78, 79], which include positive feelings (happiness, interest), and negative emotions (anger, sadness), using the 20-item Positive and Negative Affect Scale (PANAS) [90]. Similarly, The Profile of Mood States (POMS) asks about experiences of 65 different emotions, including some positive items, such as “lively” and “relaxed” [91], and was used in one study [20]. The General Health Questionnaire (GHQ) was used in 14 studies [5, 17, 19, 23–25, 38, 45, 47, 49, 54, 58, 60, 73]. It contains some positively worded items (“In the last 2 weeks I have… been able to concentrate”, “felt I have been playing a useful part” and “feeling reasonably happy”) but was designed and validated as a screening tool for psychiatric disorders, with higher scores indicative of greater distress [92]. Other studies which measured on poor mental health were excluded from this review.

Full details of the included studies are presented in Table 2, which is ordered by greenspace characteristic. Where articles cover multiple characteristics, the study appears under multiple headings.

### Greenspace characteristics

#### Amount of local-area greenspace

21 studies examined associations between quantities of local-area greenspace and mental wellbeing, 2 of which were longitudinal. Most calculated the proportion of greenspace for each Lower-Layer Super Output Area (LSOA, a geographic area generated for being as consistent in population size as possible, with a minimum population of 1000 and the mean of 1500), Census District (CD), or within a defined radius of residents. Two articles measured greenspace area per capita. Of 15 studies, one was restricted to public greenspace [39], and 14 included only urban areas.

Only four (cross-sectional) studies measured hedonic and eudaimonic mental wellbeing (SWEMWBS and ONS4). No statistically significant association was reported between greenspace and mental wellbeing in three studies [46, 52, 72], although urban residents who reported “sufficient local greenspace” showed significantly higher SWEMWBS scores [52]. However, Wood et al.’s study found that a 1ha increase in park area within a 1.6km walk of an individual’s home showed a 0.070-point increases in SWEMWBS score [53]; this suggests that examining greenspace around individuals, rather than aggregating to local area, may better detect associations.

Five studies, 4 of which were Good quality and based in urban areas, found that life satisfaction was significantly higher in areas with more greenspace [24, 39, 41, 51], albeit with small linear effect sizes of 0.002–0.003 [24, 39]. The study by White et al. included a large sample, over 10,000 individuals, demonstrating a slight but significant association between LSOA greenspace proportions and life satisfaction. Another large study by the same authors found no significant association between mental wellbeing and the amount of rural local-area greenspace [25], suggesting that associations may differ between urban and rural environments.

An ecological analysis of over 172 countries measured the amount of green land cover per km^2^, adjusted for the nation’s size, finding a significant association with better life satisfaction. Despite the large sample size and strong odds ratios (2.450), the study was of poor methodological quality, due to its ecological design and hence inability to adjust for individual-level confounding [51]. Four studies also found the quantity of urban greenspace was associated with quality of life or mental health, characterised by the SF-36 scale and its sub-components [41–43, 74]; however, three others, which included only public urban greenspace, found no association [39, 44, 50]. Taylor et al. observed mixed results: the amount of urban greenspace was positively and significantly associated with hedonic wellbeing for two cities in Australia, but not two others in New Zealand [48].

Based on these Good quality studies, we conclude that there is adequate evidence for an association between local-area urban greenspace and life satisfaction, but not rural greenspace. Mixed results provide inadequate evidence for associations with quality of life, mental health, and multi-dimensional mental wellbeing.

GHQ was the outcome in 8 studies, of which 6 were Good quality and 3 were confined to urban areas. All but one [45] found an inverse association between the amount of greenspace and GHQ score [5, 17, 23–25, 47, 49, 50], implying reduced mental distress; again, linear regression coefficients varied considerably, from 0.003 to 0.431. The Fair quality study by Dzhambov et al., however, found no statistically significant association for objective greenspace quantities, but observed significantly lower GHQ scores for those with higher perceived greenness in their neighbourhood [45]. In a longitudinal study, Alcock et al. found that people moving to areas with higher greenspace proportions had significantly lower GHQ score after relocating, averaging 0.430 points lower 3 years post-move [23]. Therefore, there was adequate evidence for the inverse association between the amount of local-area greenspace and (lower) GHQ score.

#### Greenspace types

A total of 8 Good and 2 Fair quality studies classified greenspace according to greenspace types, using bespoke classification systems; no consensus was observed regarding greenspace typology. Four of these were longitudinal studies.

Only one Fair study measured hedonic and eudaimonic wellbeing, with WEMWBS, comparing linear associations between the amount of sport, recreational and ‘natural’ spaces within a 1.6km buffer of the individual [53]. The strongest associations were observed for sports (0.430 increase in WEMWBS for each additional space), followed by recreational and natural spaces (0.110 each).

One research group conducted four studies (3 longitudinal) using the longitudinal Swedish Health Survey (SHS), based in suburban and rural areas. They classified public greenspace within 300m of each residents’ home into 5 aspects: Serene (quiet, audible ‘nature’), Wild (undeveloped, no visible human impact), Lush (biodiversity), Spacious (large cohesive area) and Cultural (cultural heritage, old trees) [18, 54, 58]. Two studies measured GHQ: the first found associations between Serene or Spacious greenspace and slightly, but significantly, lower GHQ scores for physically active individuals; however, associations with Spacious greenspace held only for women [54]. In the second, only women moving to areas with Serene greenspace had significantly lowered GHQ scores, but with much higher odds than in Annerstedt et al.’s work [58]. In a cross-sectional analysis, these authors found that the total number of green aspects (Serene, Wild, Lush, Spacious, Cultural) was associated with slightly better SF-36 Vitality scores for women [18]. The third longitudinal study found marginally but significantly lower GHQ scores for greater numbers of different green aspects, including those moving between areas [60].

In a cross-sectional study, based on 12,697 observations from 2,020 residents of rural England, no association was found between LSOA land cover classes and GHQ scores. However, individuals who relocated to areas with more arable, grass, ‘natural’, mountainous and heath land had significantly lower GHQ scores post-move [38].

Among 3 cross-sectional studies, urban residents with higher amounts of local vegetative or ‘natural’ greenspaces reported better mental wellbeing: vegetation density and cover, from field surveys and satellite imagery in Australia, were strongly and significantly associated with life satisfaction [55]. The number of trees, or an indicator of how ‘green’ the neighbourhood is, were significantly associated with better mental health (SF-36 Mental Component) and life satisfaction [57, 59]. Residents’ ratings of the ‘quality of their local natural environment’, on a scale of 0–10 (very dissatisfied to very satisfied), was associated with higher SF-36 Mental Component Summary scores [59].

A large cross-sectional study in the UK used app data on users’ self-reported feelings, while their phones’ GPS linked their location to a land-cover database; this novel study therefore benefits from measuring happiness in situ. Being in mountainous, woodland or ‘semi-natural grassland’ areas, as opposed to urban, was associated with approximately 2-points higher happiness, on a scale of 0–10, although no additional factors were controlled for [56].

While most of these studies were Good quality, interpretation is difficult due to lack of consensus in greenspace classification; in addition, four reports were based on data from the same survey. All but one were restricted to either urban or rural areas, so comparisons between these environments is not possible; however, larger effect sizes were observed in rural studies. Two of the Swedish studies concluded that green aspects were associated with lower GHQ scores for women, while 6 others highlighted that Serene (quiet, ‘natural’) and ‘natural’ rural greenspaces were associated with improved life satisfaction, SF-36 and lower GHQ scores, although none defined the term ‘natural’. Additionally, two studies reported an association between subjective perceptions of local greenspace and mental wellbeing. Evidence is therefore limited.

#### Visits to greenspace

Seventeen papers reported studies of visits, either comparing mental wellbeing scores before and after an intervention (*n* = 7), or testing cross-sectional associations with greenspace visiting patterns (*n* = 10).

Fair quality studies compared happiness and positive affect for those walking in ‘natural’ versus indoor environments [69], and walks in urban versus green areas [66]. The former reported a statistically significant difference in favour of greenspace walking, the latter did not. In a further Fair quality cross-sectional study, Marselle et al. reported a positive association between perceived restorativeness of the walking environment and positive affect and happiness [67].

Duvall and Kaplan observed 73 individuals on a wilderness expedition; attention and affect were improved post-expedition, persisting for 3–4 weeks [62]. Although effects were quite large (score changes of 0.270 to 0.340), participants were not blind to the intervention. A Fair quality uncontrolled study encouraged individuals to engage with ‘nature’ for 30 days by noticing/protecting wildlife, sharing experiences, or connecting with ‘nature’. Participants reported greater happiness following the programme [71]. Similarly, Molsher and Townsend noted mental wellbeing improvements following engagement with environmental volunteering projects [68], although their study displayed high risk of bias. Jakubec, however, reported no association between visiting greenspaces and Quality of Life Inventory score, in a Poor quality study [64].

A further 10 cross-sectional studies of varying quality examined self-reported greenspace visit frequency. Three studies measured both hedonic and eudaimonic wellbeing, with mixed findings [61, 63, 72]. In the first Fair study, university students who claimed greater typical contact with nature reported better mental wellbeing using Ryff’s Scale of Psychological Wellbeing [63, 93]. These findings were not replicated in a Good study by Glichrist et al., who examined associations between mental wellbeing (SWEMWBS) and greenspaces surrounding workplaces in Scotland [61]. White et al.’s Good study, measuring ONS4, found that those spending time outdoors and in nature every day, compared to never, had strong odds (OR 1.960) of a high sense of worth, the effect size decreasing with visit frequency. No associations were detected for visit frequency and hedonic wellbeing, although those reporting visiting greenspace the previous day had higher happiness scores, with no associations for life satisfaction, anxiety or worth [72].

A further 5 studies, one of which was Poor, showed that quality of life and mental health were improved, and GHQ scores reduced, with the number of greenspace visits [19, 21, 50, 52, 65]; Triguero-Mas et al. also noted that associations with mental health were stronger for males than females [50] In a Good study, Mitchell found that those who more regularly visited a local park had lower GHQ scores [19]. However, although Panno et al. observed that greater frequency of greenspace visits was associated with higher hedonic wellbeing, these results were not statistically significant [70], and Dzhambov et al. found no association between time spent in greenspace and GHQ [45].

Due to the mixed quality and inconsistent results, evidence for an association between greenspace visit frequency and mental wellbeing is considered limited.

#### Views of greenspace

Association between views of greenspace and mental wellbeing was reported in 3 papers. Gilchrist et al.’s Good quality study found that workers’ satisfaction with their office views, particularly of trees, lawns and flowering plants, was associated with improved mental wellbeing (SWEMWBS) scores [61]. Similarly, urban residents reporting greater visibility of trees from their home had slightly better life satisfaction [59]. Pretty et al. observed increases in self-esteem for those viewing rural pleasant scenes, while both unpleasant urban and rural scenes could be detrimental; however, they did not control for potentially confounding factors [20]. The mixed quality and small study sample leads us to classify the evidence here as inadequate.

#### Greenspace accessibility

We identified 8 cross-sectional studies, mostly Good quality, which tested associations between greenspace accessibility and mental wellbeing. Two studies measured mental health using the SF-12 Mental Component, with significant positive findings [57, 74]. In the first, a weak association was found with Euclidean (direct) distance from homes to the nearest public greenspace [74]. In the second, Sugiyama et al. used the Neighbourhood Environment Walkability Scale, which measures self-reported greenspace access. Access to the highest of levels of greenspace (perceived neighbourhood greenness, terciles) was associated with strong odds (OR 1.270) of better mental health [57].

Only one, Fair study compared public greenspace within different Euclidean buffers around individuals’ postcodes [49]. Triguero-Mas et al. found greater amounts of greenspace within 300m were significantly associated with reduced risk of high GHQ scores (dichotomised around 3), with consistent results for control buffers of 100m, 500m, and 1km [49]. Bos et al. found that greenspace within 3km, but not 1km, of homes was significantly associated with greater quality of life [43], although this study was rated as Poor study because of limited statistical reporting. In a larger study, scores on the SF-36 Vitality scale were associated with rural greenspace, but this was only significant for women and within 300m (but not 100m), of their home [18]. Maas et al.’s large cross-sectional study showed that those with more greenspace within 1km, but not 3km, had slightly lower GHQ scores, contrary to findings by Bos et al. [43, 47]. Dadvand et al. also measured GHQ (dichotomised around 3), finding strong odds of low GHQ scores for the amount of greenspace within 100m of homes (OR 1.320), effect sizes reducing with distance (OR 1.250 for 250m, 1.170 for 500m); stronger associations were also noted for subjective, than objective, proximity to greenspace, measured as self-report and calculated presence of a greenspace within a 10-minute walk [73]. Dzhambov et al. also found a significant association between subjective accessibility (time to walk to nearest greenspace) and lower GHQ, although associations for objectively measured Euclidean distance were not statistically significant [45].

Although several of these studies reported an association between greenspace accessibility and aspects of mental wellbeing, different measures of both were used and findings were inconsistent, providing limited evidence of an association.

#### Subjective connectedness to nature

We identified 7 cross-sectional studies examining associations between subjective connection to nature and mental wellbeing. The Connectedness to Nature Scale measures the extent to which individuals ‘feel nature is part of their identity’, with particular emphasis on sense of care for nature; this has been linked to the theory of biophilia: that humans possess an innate desire to affiliate with other forms of life [3, 69]. Of these studies, 5 were of Poor quality, with no controls for potential confounding. Four studies demonstrated that self-reported ‘connection to nature’ was positively associated with mental wellbeing [76–79]. Effect sizes were moderate and consistent across the studies, although lower methodological quality means their results have limited generalisability; only one was of Good quality, and adjusted for potentially confounding factors. Similarly, meaning in life, quality of life, happiness and affect were higher for those who reported greater connection to nature [65, 75, 76, 79]. Life satisfaction was also positively related to nature connectedness in two studies [79, 80], with moderate effect sizes, although Zhang et al. revealed that the association only held for those who actively engaged with nature [80]. While consistent in their findings, poor study quality means that the evidence is inadequate.

## Discussion

### Summary of findings

While both the World Health Organisation and United Nations agree that greenspace is vital for healthy, liveable environments [11, 12], it remains unclear which amounts, types and uses of greenspace are most beneficial to mental wellbeing. Previous reviews have focussed on associations between greenspace (or nature) and general health or mental distress [7, 8, 12, 31], but we are not aware of any previous systematic reviews of published evidence specifically for associations between greenspace and validated, positive measures of mental wellbeing in adults. Even after stratifying our review according to the six main ways in which greenspace was conceptualised and measured, methodological heterogeneity precluded meta-analysis. We therefore undertook a narrative synthesis.

The largest number of studies were concerned with the amount of local-area greenspace, although few used detailed hedonic and eudaimonic wellbeing measures. Consistent results revealed adequate evidence for an association between urban local-area greenspace and life satisfaction. This result did not hold for rural greenspace, however. There was also adequate evidence for an association between local-area greenspace and lower GHQ scores.

Inconsistencies in the categorisation of greenspace types, and dearth of definitions, made it difficult to synthesise results; limited evidence was therefore found for associations between mental wellbeing and variety and ‘nature’ in land cover. Evidence was similarly limited for greenspace accessibility, with results generally concluding that nearer greenspace has the strongest associations, but with results differing according to the mental wellbeing measure; limited evidence was also found for associations between greenspace visits and mental wellbeing.

However, while there was some evidence for an association between mental wellbeing and views of greenery and connectedness to nature, this was considered inadequate, due to the mixed quality and small sample sizes of studies. Table 4 provides full details of the evidence summary and implications for research and policy.

**Table 4:** Summary of findings and implications.

*Greenspace Characteristic*	*Summary of Evidence*	*Strength of Evidence*	*Implications*
Amount of local area greenspace	Positive association between urban greenspace and life satisfaction	Adequate	*Research*: Studies are required to measure both hedonic and eudaimonic wellbeing Associations may differ between urban and rural environments National studies should stratify for urban/rural setting Local-area statistics may be less effective at detecting associations than measures which consider greenspace relative to the individual. Greenspace within set distances of individuals should be further investigated. Methods should consider the potential spatial nature of the data More longitudinal analyses are required to establish causality Greenspace measures should consider where people spend their time (ie while commuting, at work), not just relative to homes *Policy*: Increasing provision local-area greenspace in urban environments is recommended for potential benefits to life satisfaction
	Inverse association between urban greenspace with GHQ	Adequate	*Research*: Studies are required to measure positive mental wellbeing (both hedonic and eudaimonic dimensions) *Policy*: Increasing provision of urban local-area greenspace is recommended for potentially reducing symptoms of psychiatric distress
Greenspace types	Some association between ‘nature’/variety in land cover and aspects of mental wellbeing	Limited	*Research*: Studies are required to measure both hedonic and eudaimonic wellbeing More consistency is needed in establishing a greenspace typology Specific features of greenspace should be investigated More consistency is needed in defining terms, particularly ‘nature’, which is often undefined Measures of greenspace quality should also be included *Policy*: Variety and nature in greenspace types may be important, but currently more evidence is required to recommend this for mental wellbeing benefit
Visits to greenspace	Frequency of visits to greenspace may be associated with aspects of mental wellbeing	Limited	*Research*: Studies are required to measure both hedonic and eudaimonic wellbeing More objective assessments of greenspace visiting patterns are required Social context and individual experiences of greenspace patterns should be considered Participants must be blind to interventions to ensure a fair sample More controlled case studies, and longitudinal analyses may help in understanding the direction of associations *Policy*: Promoting visits to greenspace may improve aspects of mental wellbeing, though more evidence is required
Views of greenspace	Views of greenspace/green features may be associated with some aspects of mental wellbeing	Inadequate	*Research*: Studies are required to measure both hedonic and eudaimonic wellbeing Much more research should examine associations between views of greenspace and mental wellbeing With potential differences between views from homes and workplaces, greenspace measures should consider where people spend their time
Greenspace accessibility	Greenspace closer to homes may be most strongly associated with aspects of mental wellbeing	Limited	*Research*: More studies are required to measure both hedonic and eudaimonic wellbeing Accessibility measures need greater consistency, including controlled sensitivity analyses Network, rather than just Euclidean distances, should be applied Social and physical barriers to access should be considered Quality and facilities of greenspaces require further investigation and consistency, for example use of the Green Flag Award for parks Spatial Methods Thorough testing of Government guidelines is necessary to provide robust evidence of mental wellbeing benefit *Policy*: There is currently a lack of evidence recommend the guideline of providing greenspace within a 300m buffer specifically, for mental wellbeing in particular
Subjective connection to nature	Personal connection to nature may be associated with mental wellbeing	Inadequate	*Research*: Studies must control for potentially confounding factors More objective assessments of connection to nature and mental wellbeing are required More consistency is needed in defining terms, particularly ‘nature’, which is often undefined

### Mental wellbeing measures

Only 14 of the 52 studies used a measure of mental wellbeing that captured both hedonic and eudaimonic dimensions, while others measured aspects such as life satisfaction, happiness and quality of life. GHQ, which was designed as a psychiatric screening tool, was included as a prevalent surrogate in the literature, which includes some positive items. Papers using other psychiatric screening tools were excluded if they covered only symptoms, ie mental distress.

### Greenspace definitions and indicators

We identified 6 types of assessment in greenspace studies: amount of local-area greenspace, greenspace types, visits to greenspace, views of greenspace, greenspace accessibility and self-reported connection to nature.

The amount of local-area greenspace was most commonly measured as the proportion of greenspace in a resident’s local area, or more specifically within a set radius of participants’ homes. Most of these studies were restricted to urban areas. Most researchers quantified greenspace objectively, while a small number of studies reported associations with perceptions of the adequacy of the amount of local greenspace provision. All studies used either linear or logistic regression, which may overestimate associations in spatial data. Although a number of studies examined different types of greenspace, no consensus was observed for a typology, and as such conflicting results were observed.

One of the UN’s Sustainable Development Goals is to “provide universal access to…green and public spaces” [11]; most studies assessed accessibility by distance to greenspace. While the EU and UK recommend that individuals should have access to a greenspace within 300m of their home [14, 94], only one study conducted sensitivity analysis to test this guideline [49]; no difference in associations was observed for buffers of 100m, 300m, 500m and 1km. One study used buffer radii of 100m and 300m, reporting a significant association between the latter and mental wellbeing, while a second found that associations with GHQ decreased with distance, at 100m, 250m and 500m buffers. Others found contradictory results using radii of 1 and 3km. Another drawback was the use of Euclidean distance, which doesn’t account for access routes. Application of network distance and consideration of pedestrian routes may give a greater indication of accessibility on foot.

Greenspace visiting patterns were measured inconsistently, in small or cross-sectional studies. Individuals who visited greenspace more often reported greater mental wellbeing, though a second study found this held only for eudaimonic wellbeing; no associations were found in an analysis of greenspace adjacent to workplaces. This study did however report a positive association with views of greenspace from the workplace. This is in keeping with previous research showing that green views reduce the effects of stress [8–10, 95]. While two studies highlighted that the perceptions of greenspace quality were more strongly associated with mental wellbeing than quantity [52, 59], the size of this difference was not estimated.

Individual connection to nature, assessed in seven studies, relied on self-report for both the greenspace and wellbeing measures, thereby carrying a high risk of reporting bias, especially since few controlled for potentially confounding factors.

### Strengths and limitations

We conducted a comprehensive database search, thorough screening of articles, risk of bias assessment, and detailed narrative synthesis of the 50 studies which met our inclusion criteria. We identified six different ways in which greenspace was conceptualised and measured, and by which we stratified our review. We believe this is the first review to systematically appraise the evidence for associations between greenspace and adult mental wellbeing, using only validated measures of positive mental health.

Selection criteria were designed to ensure results of sufficient quality and relevance, and we consulted an information specialist to maximise search efficiency. Screening was undertaken by two independent reviewers, to minimise potential bias. While our criteria were designed to be inclusive, an element of subjectivity means there was a possible risk of excluding potentially interesting studies; we attempted to minimise this by appraising each study with assessments recommended by the Cochrane Handbook, which provides guidance for internationally recognised highest-standard research [32, 34–37].

We considered all greenspaces, not restricting our criteria to studies specifically in urban areas, although some studies were confined to urban or rural locations. Nationwide studies were likely to have included both, without stratifying for setting. It was difficult, therefore, to draw clear conclusions about interactions between urban and rural location and associations with mental wellbeing. Although there is interest in understanding how urban greenspaces should best be designed and constructed, it was not possible to draw conclusions specifically for those living in cities.

Only one-quarter of included studies measured both hedonic and eudaimonic mental wellbeing; the majority focused only on aspects such as life satisfaction, affect and vitality, while others used measures (such as the GHQ) which combined positive and negative (distress) items.

While several studies implied that ‘nature’ was associated with aspects of mental wellbeing, none provided a definition of this term. To further complicate matters ‘nature’ and ‘greenspace’ were sometimes used synonymously [17–19, 21, 72]. Vegetative or ‘natural’ greenspaces, such as those described as ‘serene’ (quiet, ‘natural’), or with more trees, were most strongly associated with aspects of mental wellbeing, although one study found a stronger association for sports facilities. However, there were few direct comparisons between greenspace types. While Government Guidance provides a standardised greenspace typology for urban planning in the UK [15], no studies used this classification.

Studies that considered greenspace accessibility were limited to estimates of Euclidean distances from home rather than access routes [96]. These studies did not take account of participants’ routines, or where they spent their time. None of the included studies assessed greenspace quality (such as captured by the Green Flag Award [97], or the social contexts in which greenspaces are situated [7, 98].

Only 6 out of 50 papers reported longitudinal studies. Cross-sectional analyses cannot distinguish between reverse causality and associations which may be causal in nature, and, like all observational studies, are prone to confounding (especially by indication) and bias. Although 26 studies were deemed to be of Good quality, 12 were Fair, and the remaining 12 were Poor; this was mostly due to lack of control for potentially confounding, minimal statistical reporting, and, in 3 cases, lack of participant blinding to an intervention.

## Conclusions

We sought to synthesis and appraise the evidence for associations between greenspace and mental wellbeing, but found few studies measuring both hedonic and eudaimonic wellbeing. Results suggest associations between greenspace and mental wellbeing, particularly hedonic wellbeing. We discovered adequate evidence for associations between urban greenspace and life satisfaction; however, the evidence for the remainder of the greenspace characteristics, including greenspace (land use) type, accessibility, viewing and visiting patterns, was limited or inadequate. Although not a true measure of mental wellbeing, studies using the GHQ were prevalent in the literature. This measure includes some positive items, and we further concluded that there was adequate evidence for associations between greenspace and lower GHQ scores. While our review was limited by the lack of available data to conduct a meta-analysis, we were able to highlight key areas for future research through our narrative synthesis.

Government guidelines for greenspace provision require robust evidence, but evidence is currently not sufficient for informed, specific planning recommendations. Further methodological work in this field is needed, including the development of operational definitions of ‘nature’ and ‘natural’, and agreement on a land use typology. Measures of greenspace quality are also needed. More studies are required to measure both hedonic and eudaimonic mental wellbeing. Greenspace accessibility should also be measured more specifically, using individual travel distances, using spatial methods of analysis, to better understand how greenspaces should be designed and incorporated into environments. Further research is needed that considers differences in associations between greenspace and mental wellbeing in urban versus rural settings.

## Supporting information

S1 Table Heatmap of risk of bias for studies evaluated using the Newcastle-Ottawa Scale adapted for cross-sectional studies. (DOCX)

S2 Table Heatmap of risk of bias for studies evaluated using the Cochrane RoB 2.0 tool. (DOCX)

S3 Table PRISMA 2009 checklist. (DOC)
