# Supplementary material for: The relationship between greenspace and the mental wellbeing of adults: A systematic review
Source: PLoS One. 2018 Sep 12;13(9):e0203000. doi: 10.1371/journal.pone.0203000 (PMC6135392; doi:10.1371/journal.pone.0203000)
Supplement: S1 Table — (DOCX) [file pone.0203000.s001.docx]

|  | Alcock et al. 2015 | Alcock et al. 2014 | Ambrey and Fleming 2014 | Ambrey 2016a | Ambrey 2016b | Ambrey 2016c | Annerstedt et al. 2012 | Astell-Burt et al. 2014 | Bos et al. 2016 | Bjork et al. 2008 | Cervinka et al. 2012 | Dadvand et al. 2016 | De Vries et al. 2003 | De Vries et al. 2013 | Dzhambov 2018 | Gilchrist et al. 2015 | Herzog and Stevey 2008 | Houlden et al. 2017 | Howell et al. 2011 | Howell et al. 2013 | Kamitsis and Francis 2013 | Krekel et al. 2015 |
| --- | --- | --- | --- | --- | --- | --- | --- | --- | --- | --- | --- | --- | --- | --- | --- | --- | --- | --- | --- | --- | --- | --- |
| **Selection** |  |  |  |  |  |  |  |  |  |  |  |  |  |  |  |  |  |  |  |  |  |  |
| Representativeness of the sample |  |  |  |  |  |  |  |  |  |  |  |  |  |  |  |  |  |  |  |  |  |  |
| Sample size |  |  |  |  |  |  |  |  |  |  |  |  |  |  |  |  |  |  |  |  |  |  |
| Non-respondents |  |  |  |  |  |  |  |  |  |  |  |  |  |  |  |  |  |  |  |  |  |  |
| Ascertainment of exposure |  |  |  |  |  |  |  |  |  |  |  |  |  |  |  |  |  |  |  |  |  |  |
| **Comparability** |  |  |  |  |  |  |  |  |  |  |  |  |  |  |  |  |  |  |  |  |  |  |
| Comparable outcome groups |  |  |  |  |  |  |  |  |  |  |  |  |  |  |  |  |  |  |  |  |  |  |
| **Outcome** | | | | | | | | | | | | | | | | | | | | | | |
| Ascertainment of the outcome |  |  |  |  |  |  |  |  |  |  |  |  |  |  |  |  |  |  |  |  |  |  |
| Statistical test |  |  |  |  |  |  |  |  |  |  |  |  |  |  |  |  |  |  |  |  |  |  |

**S1 Table. Heatmap of risk of bias for studies evaluated using the Newcastle-Ottawa Scale adapted for cross-sectional studies**

|  | Luck et al. 2011 | Maas et al. 2009 | Mackerron and Mourato 2013 | Marselle et al. 2013 | Mitchell 2013 | Nisbet ET AL. 2011 | Panno et al. 2017 | Sugiyama ET AL. 2008 | Taylor et al. 2017 | Triguero-Mas et al. 2015 | Triguero-Mas et al. 2017 | Van den Berg et al. 2016 | Van den Bosch et al. 2015 | Vemuri and Costanza 2006 | Vemuri et al. 2011 | Ward Thompson et al. 2014 | Weimann et al. 2015 | White et al. 2014 | White et al. 2017 | Wood et al. 2017 | Zelenski and Nisbet 2014 | Zhang et al.2014 |
| --- | --- | --- | --- | --- | --- | --- | --- | --- | --- | --- | --- | --- | --- | --- | --- | --- | --- | --- | --- | --- | --- | --- |
| **Selection** |  |  |  |  |  |  |  |  |  |  |  |  |  |  |  |  |  |  |  |  |  |  |
| Representativeness of the sample |  |  |  |  |  |  |  |  |  |  |  |  |  |  |  |  |  |  |  |  |  |  |
| Sample size |  |  |  |  |  |  |  |  |  |  |  |  |  |  |  |  |  |  |  |  |  |  |
| Non-respondents |  |  |  |  |  |  |  |  |  |  |  |  |  |  |  |  |  |  |  |  |  |  |
| Ascertainment of exposure |  |  |  |  |  |  |  |  |  |  |  |  |  |  |  |  |  |  |  |  |  |  |
| **Comparability** |  |  |  |  |  |  |  |  |  |  |  |  |  |  |  |  |  |  |  |  |  |  |
| Comparable outcome groups |  |  |  |  |  |  |  |  |  |  |  |  |  |  |  |  |  |  |  |  |  |  |
| **Outcome** | | | | | | | | | | | | | | | | | | | | | | |
| Ascertainment of the outcome |  |  |  |  |  |  |  |  |  |  |  |  |  |  |  |  |  |  |  |  |  |  |
| Statistical test |  |  |  |  |  |  |  |  |  |  |  |  |  |  |  |  |  |  |  |  |  |  |

Key:

Low RoB

Some Rob
