# Supplementary material for: The relationship between greenspace and the mental wellbeing of adults: A systematic review
Source: PLoS One. 2018 Sep 12;13(9):e0203000. doi: 10.1371/journal.pone.0203000 (PMC6135392; doi:10.1371/journal.pone.0203000)
Supplement: S2 Table — (DOCX) [file pone.0203000.s002.docx]

**S2 Table. Heatmap of risk of bias for studies evaluated using the Cochrane RoB 2.0 tool**

|  | Duvall and Kaplan 2014 | Jakubec et al. 2016 | Marselle et al. 2015 | Molsher and Townsend 2016 | Nisbet and Zekenski 2011 | Pretty et al. 2005 | Richardson et al. 2016 |
| --- | --- | --- | --- | --- | --- | --- | --- |
| **Bias arising from randomisation process** |  |  |  |  |  |  |  |
| Was the allocation sequence random? | PN | PN | PN | N | PY | N | NI |
| Were there baseline imbalances that suggest a problem with the randomisation process? | NI | NI | NI | NI | NI | NI | NI |
| **Bias due to deviations from intended interventions** |  |  |  |  |  |  |  |
| Were participants aware of their assigned intervention? | Y | Y | Y | Y | PN | PN | PN |
| Were personal aware of the participants’ assigned intervention? | Y | Y | Y | Y | PY | PY | NI |
| **Bias due to missing outcome data** |  |  |  |  |  |  |  |
| Was the outcome data available for all, or nearly all, participants randomised? | PY | PY | PY | PY | PY | PY | PY |
| Are the proportions of missing outcome data and reasons for missing outcome data similar across groups? | NI | PY | Y | NI | NI | NI | PY |
| **Bias in measurement of the outcome** |  |  |  |  |  |  |  |
| Were outcome assessors aware of the intervention received by study participants? | Y | Y | Y | Y | Y | Y | Y |
| **Bias in the selection of the reported result** |  |  |  |  |  |  |  |
| Are reported outcome data likely to have been selection, on the basis of results, from… multiple outcomes? | PN | PN | PN | PN | PN | PN | PN |
| … multiple analyses of the data? | PN | PN | PN | NI | PN | PN | PN |

Y = Yes

PY = Possibly Yes

PN = Possibly No

No = No

NI = No Information

High RoB

Some Concerns of RoB

Low RoB

No Information
